# Reduction in inhibitory control is sufficient to generate hyperalgesia in a spiking model of nociceptive integration in the superficial dorsal horn

**DOI:** 10.1186/1471-2202-14-S1-P1

**Published:** 2013-07-08

**Authors:** Mafalda Sousa, Peter Szucs, Paulo Aguiar

**Affiliations:** 1 Faculdade de Medicina da Universidade Porto, Porto 4200 -319, Portugal; 2 Instituto de Biologia Molecular Celular, Porto 4150-180, Portugal; 3 Centro de Matemática da Universidade Porto, Porto 4169-007, Portugal

## 

The spinal cord's dorsal horn is a major termination site for primary afferents carrying sensory information from the periphery. The superficial dorsal horn (SDH, laminae I and II), in particular, receives inputs from nociceptive C and Aδ fibers, and have an important role in relaying and processing nociceptive information. This work focuses on the pain condition named hyperalgesia, characterized by an increased response to a stimulus that is normally painful. Much is still unknown regarding the central mechanisms giving rise to this condition.

Here we present a large network spiking model for hyperalgesia which includes significant functional components of the nociceptive circuit in the SDH. The network architecture (Figure 1A) was defined according to key experimental results, namely: the somatotopic mapping between body surface and the superficial dorsal horn [1]; the presence of an inhibitory control which, under normal conditions, leads to a proper perception of pain [2]; the existence of divergent/convergent fiber inputs to lamina I (LI) neurons [3]; and the presence of inhibitory interneurons, i.e. islet cells, with inputs/outputs within lamina II (LII) [4]. In the model, the connectivity profile between LII interneurons (excitatory, Exc and inhibitory, Inh) and LI projection neurons creates a mexican-hat input profile with a strong centered excitation flanked by a surrounding inhibition. Activity level control was provided by feed-forward inhibition from the inhibitory interneurons in LII. The network model was created and simulated using the simulation environment NeuralSyns [http://sourceforge.net/projects/neuralsyns/]. Neurons were represented as integrate-and-fire units with conductance based synapses. The full model was comprised of 2.600 neurons and 320.000 synapses. The analysis was performed by assessing the changes produced in the receptive fields of LI nociceptive specific projection neurons (NS), both in normal (control) conditions and after reduction in inhibitory control from LII inhibitory interneurons (Figure 1B). Fiber activity (Aδ, C) was modelled using spatially modulated Poisson activations. The receptive field associated with reduced inhibitory control occupies a larger skin area and involves higher mean firing rates. These two results correlate respectively with secondary and primary hyperalgesia.

**Figure 1:**
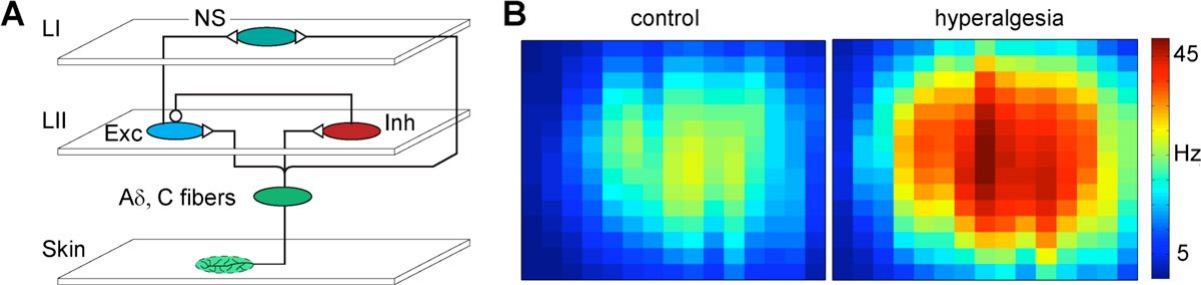
**A) Network model architecture**. B) Receptive field of a lamina I neuron - mean firing rates associated with a skin area with 15 × 15 length units. Data filtered with a Gaussian kernel, σ = 2 length units.

## Conclusions

This model shows that mechanisms interfering with the balance between excitation/inhibition and reducing, temporarily or chronically, the feed-forward inhibitory control in the SDH have the potential to give rise to hyperalgesia. Understanding how this pain condition occurs provides important information on how to reverse pathological situations. This work was supported by grant SFRH/BD/60690/2009 from FCT.
